# Cytotoxicity and Antibiofilm Activity of Silver-Polypropylene Nanocomposites

**DOI:** 10.3390/antibiotics12050924

**Published:** 2023-05-17

**Authors:** Denise Bellisario, Loredana Santo, Fabrizio Quadrini, Maryam Hassiba, Nour Bader, Shazeda H. Chowdhury, Mohammad K. Hassan, Susu M. Zughaier

**Affiliations:** 1 Faculty of Economics, Universitas Mercatorum, 00186 Rome, Italy; 2 Department of Industrial Engineering, University of Rome Tor Vergata, 00133 Rome, Italy; loredana.santo@uniroma2.it (L.S.); fabrizio.quadrini@uniroma2.it (F.Q.); 3 College of Medicine, QU Health, Qatar University, Doha 2713, Qatar; 4 Center for Advanced Materials, Qatar University, Doha 2713, Qatar

**Keywords:** Ag nanocomposites, antibiofilm activity, metallic silver nanoplates, cells viability measurement

## Abstract

The development of biocompatible nanomaterials that interface with human skin and tissue is critical for advancing prosthetics and other therapeutic medical needs. In this perspective, the development of nanoparticles with cytotoxicity and antibiofilm properties and biocompatibility characteristics are important. Metallic silver (Ag) exhibits good biocompatibility, but it is often challenging to integrate it into a nanocomposite without compromising its antibiofilm properties for optimal applications. In this study, new polymer nanocomposites (PNCs) with ultra-low filling content (0.0023–0.046 wt%) of Ag nanoplates were manufactured and tested. The cytotoxicity and antibiofilm activity of different composites with polypropylene (PP) matrix were examined. At first, PNCs surface were analyzed by means of AFM (atomic force microscopy) with phase contrast evaluation and FTIR (Fourier-transform infrared spectroscopy) to study the Ag nanoplates distribution. Subsequently, the cytotoxicity and growth properties of biofilms were assessed by MTT assay protocol and detection of nitric oxide radicals. Antibacterial and antibiofilm activities were measured against Gram-positive bacteria (*Staphylococcus aureus*) and Gram-negative bacteria (*K. pneumoniae*). The PNCs with silver exhibited antibiofilm activity although they did not inhibit regular planktonic bacterial growth. Moreover, the PNCs were not cytotoxic to mammalian cells and did not induce significant immune response. These features reveal the potential of the PNCs developed in this study for usage in fabrication of prosthetics and other smart structures for biomedical applications.

## 1. Introduction

In recent years, it has become increasingly important to develop biocompatible nanomaterial that interfaces with human skin and tissues for advancing prosthetics and other therapeutic medical needs. The desired nanomaterial should be devoid of cytotoxicity with minimal effect on human tissues in terms of inducing irritation, allergy, or more serious effects such as cell death and tissue necrosis [1]. Human skin and exposed tissues harbor microbiota where various microorganisms mainly nonharmful bacteria are living symbiotically.

Human skin has a robust innate defense system that prevents bacteria from invading lower layers and thereby prevent infections. However, when the protective skin layer is ruptured by injury, erosion, or burn, bacterial infections can occur [2]. Bacteria tend to form biofilms mainly on solid surfaces such as prosthetics, catheters, and impeded devices [3]. These biofilms are structured with large colonies of bacteria covered with slime composed of polysaccharides, proteins, and DNA; therefore, biofilms are more resistant to antimicrobial treatment and are very difficult to remove compared to bacteria growing in planktonic state [4]. For example, biofilm formation in urinary catheters leads to urinary tract infection and cannot be treated without removing or replacing the catheter [5]. The current status of antibiofilm on implanted prosthetics is usually treatment with antibiotics and removing or replacing the infected catheter or prosthetic. Moreover, antibiofilm material is usually used as a coating layer for medical prosthetics to prevent biofilm formation [6]. Therefore, the desired nanomaterial should have the ability to prevent or reduce biofilm formation to eliminate the risk of infection at the prosthetic and human skin or tissue interface.

The use of nanoparticles (NPs) is increasingly widespread because their small size increases the surface-to-volume ratio [7]. Moreover, the NPs of sizes in the range of 1 to 100 nm have revealed higher microbial activity [8]. Specifically, silver nanoparticles (AgNPs) have been widely studied thanks to their active role in the antimicrobial action against certain diseases that cause different infections [9,10,11]. Recently, many researchers proposed Ag-based NPs synthesized using enzymes, microorganisms, or plant extracts [12,13,14]. In fact, there are many easy and green protocols for synthesizing AgNPs from natural constituents; however, these methods have limited applications as the NPs can only be only prepared in the solution phase [15,16].

Specifically, the possibility of using and exploiting the properties of these AgNPs is limited by the ability to integrate them into polymeric nanocomposites (PNCs), whereas the possibility of adding AgNPs into polymer matrices is also a function of production method used. In particular, nowadays, hybrid thermoplastic matrices are particularly used and attractive thanks to their enhanced mechanical, thermal, electrical, optical, and magnetic properties and also to their ductility, solvent resistance, reparability, and potential recyclability [17,18,19]. Such properties are very important in biomedical devices and even more crucial is the possibility to transfer the antimicrobial and antibiofilm features of the NPs to the PNCs.

Traditionally, thermoplastic PCNs are manufactured by melt compounding in extruders, mixers, or mills. The high shear forces in melt-mixing of the thermoplastic matrix and NPs ensure greater particle separation and dispersion [20,21]. Currently, this methodology is the most pursued because it is suitable for industrial-scale processes as it is efficient and solvent-free [22,23]. Moreover, the produced PNC compound is easily processable with other molding techniques, such as extrusion or injection molding [24]. Generally, the major limitations in the production of PNC are related to technological issues. These include, for example, the difficulty to control the NPs sizes, such as the shape or the aggregation during NPs synthesis or the dispersion and integration during the PNCs manufacturing.

However, metal particles of Ag have the advantage of not being thermally degraded by the thermoplastics process temperatures (~200 °C) [25,26,27]. As a result, Walter et al. recently directly inserted the biocide agent in thermoplastics such as polyolefins [25]. Despite this, problems remain such as formation of particles clusters and the need to functionalize the metal particles to ensure better distribution within the matrix polymer [28]. This is especially true if the percentage of the filler is high, but if ultra-low percent is used, a concern would be related to the ability of the NPs to make an impact at such low concentrations.

In this study, PNCs with ultra-low metallic silver content (<0.05 wt%) as an antimicrobial agent were manufactured and tested. The cytotoxicity and antibiofilm activity of different PNCs with polypropylene (PP) matrix, and Ag nanoplates were examined. The PNCs were fabricated by the authors using a new technique called “nanocoating fragmentation” which was introduced in previous studies [29,30,31]. The cytotoxicity and growth properties of biofilms were evaluated on the surfaces of these PNCs with increasing metallic Ag content (0.0023–0.046 wt%) and compared with neat PP surfaces. The PNC containing silver possesses antibiofilm activity although it does not inhibit regular planktonic bacterial growth. Moreover, the PNC was not cytotoxic to mammalian cells and did not induce significant immune response.

## 2. Results

### 2.1. PNCs Surface Evaluation

The PNC samples used in the study were identified on the basis of mixing ratio (Ag covered PP pellet/PP pellet) percentages during the manufacturing phase. The first results useful for the study are those related to the evaluation of the surface of manufactured PNCs. Evaluating the surface conformation and the contribution of NPs on the surface is fundamental for subsequent cytotoxicity and antibiofilm assessments.

**Figure 1:**
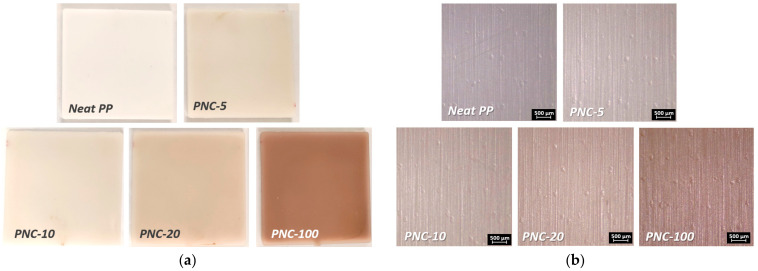
Neat PP and PNCs square molded samples. (**a**) Images of the molded samples. (**b**) Stereoscopic micrographs of the surfaces of PNCs samples and Neat sample. Note: the contents of Ag in the different samples are Neat PP (0 wt%), PNC-5 (0.0023 wt%), PNC-10 (0.0046 wt%), PNC-20 (0.0092 wt%), and PNC-100 (0.046 wt%).

The fabricated PNC samples of about 100 × 100 mm^2^ for each molding condition are shown in Figure 1a with the corresponding amounts of Ag wt% contained in the starting material used in the sample preparation.

**Figure 2:**
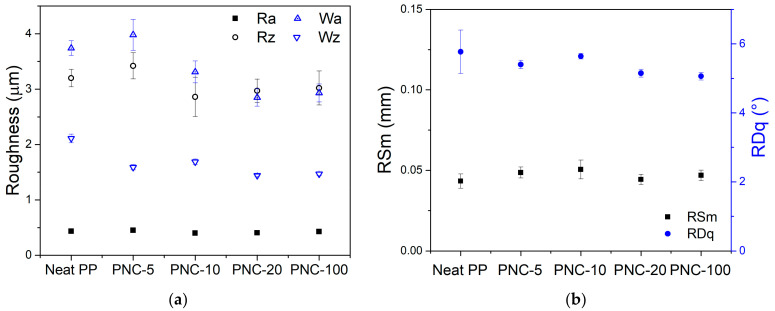
Roughness measurements: (**a**) Amplitude parameters of roughness and waviness; (**b**) spacing (RSm: mean width of the roughness profile) and hybrid roughness (RDq: root mean square slope) parameters.

Figure 1b shows the micrographic appearance of the surfaces that seems identical except for the coloring that is highlighted darker as the percentage of NPs increases. The observations of the surfaces of the different PNCs with increasing Ag wt% content were made by means of a stereoscope (Leica s9i) and the morphological characteristics were evaluated with a surface analyzer (Talysurf CLI 2000 by Taylor Hobson). All samples show an identical surface texture that is due to the mechanical processing of the steel mold used in the molding phase. A quantitative evaluation of this texturing is instead evaluated and summarized in Figure 2a,b through the roughness and waviness parameters.

The variability ranges of the amplitude parameters were narrow and limited, so as to verify that all samples were produced in the same way and that the presence of NPs did not affect, in any way, the surface morphology of PNCs. Specifically, the average roughness (Ra) varied between 0.4 and 0.45 μm, in the same way the average height of the profile elements (Rz) varied from 2.9 to 3.4 μm. These roughness amplitude values highlight a surface with non-negligible roughness levels and with a marked texture. In fact, when analyzing the waviness parameters (average waviness Wa = 2.9–3.9 μm, average height of the waviness Wz = 1.5–2 μm) significant excursions were underlined. Similarly, the spacing and hybrid parameters indicated that the mean width of the profile elements were quite similar and the root mean square slope was within the range 5°–6°.

**Figure 3:**
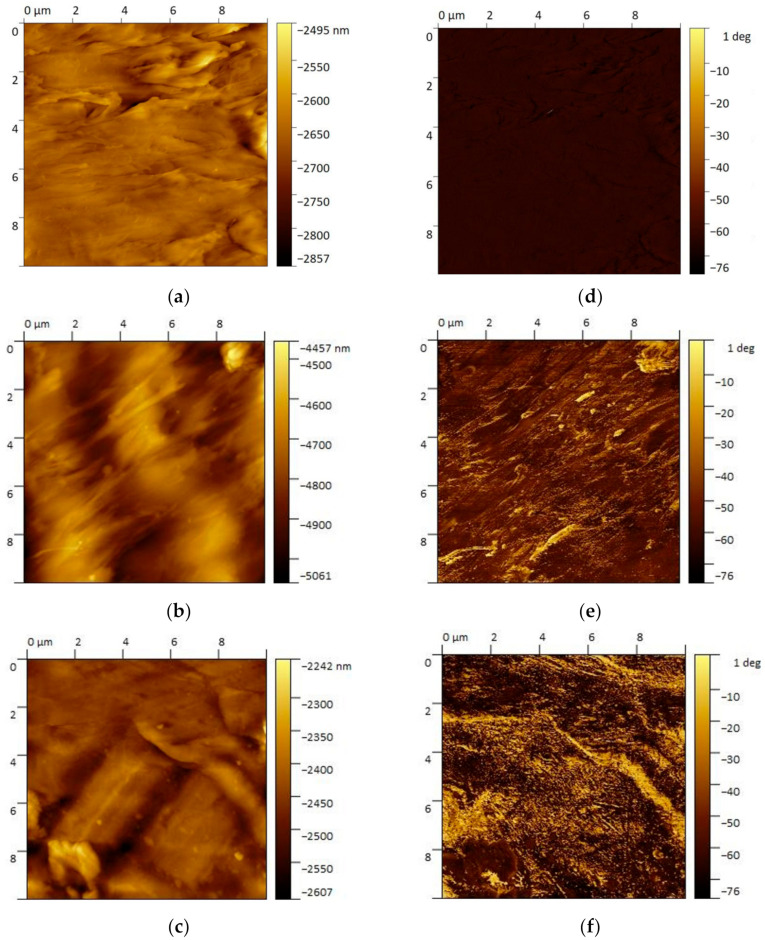
AFM height and phase images: heights (**a**) Neat PP, (**b**) PNC-10, (**c**) PNC-100 and phase (**d**) Neat PP, (**e**) PNC-10, (**f**) PNC-100. The height and phase are in the same test area.

In Figure 3a–c, AFM images are shown in order to visualize the surface morphology of the PNC samples. AFM height images clearly show the surface variability of nanocomposites. This was already highlighted by the profilometer analysis. The surface morphology is mostly related to injection molding process. In particular, this morphology is due to the surface of the mold and it differs slightly between samples. Specifically, the noticeable variability in height scales of the AFM images is primarily related to the location and the small size of the scan area. The AFM phase image (10 μm) analysis from 10 wt% and 100 wt% Ag PNC (Figure 3d,f) shows the Ag phase dispersion in the PP matrix. Specifically, metallic NPs are visible as Ag plates with nanometric thickness and a micrometric extension. The Ag nanoplates are parallel to the polymer flows during molding. In fact, the orientation of plate-like particles is strictly dependent on polymer flow [32]. Generally, particles arrange aligned near the mold surface in the injection-molding process [33]. In the PNCs, the nanoplates alignment is evident and it highlights what was already found by Sasayama et al. [34]. In particular, they observed the formation of a shell-core structure within the molded samples with microplates. Moreover, the shell layer showed a high degree of alignment of this plates.

**Figure 4:**
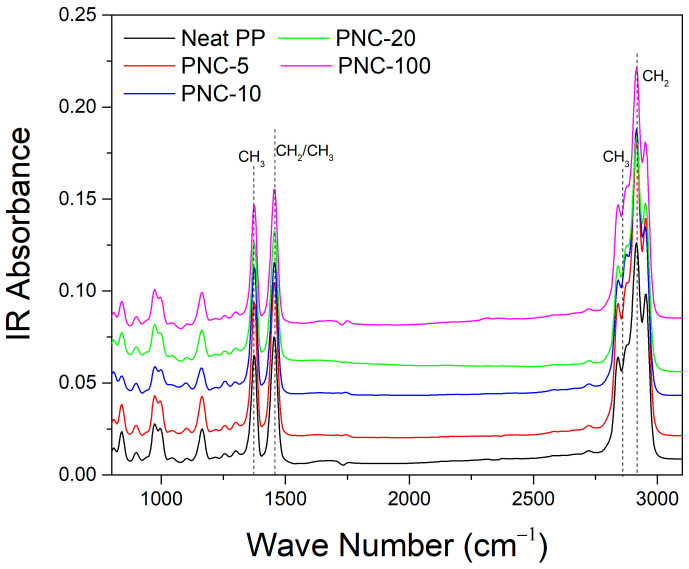
ATR-FTIR spectra for the different PNCs (the curves are vertically translated in order to better visualize the spectra).

Attenuated total reflection (ATR) Fourier-transform infrared (FTIR) spectroscopy was also used to analyze PNC samples. Figure 4 reports the absorbance spectrum in IR and it shows typical PP bands. Mainly, the bands are associated with the asymmetric elongation of the CH bonds to 2923 cm^−1^, to 1455 cm^−1^ bending vibrations ascribed to the asymmetric deformations of the group CH_2_/CH_3_, and symmetric bending vibrations attributed to the C–H vibrations of the CH_3_ group to 1370 cm^−1^. However, the bands are characteristics of PP and no significant differences between PNCs are shown. This could be related to the organic nature of the matrix and the ultra-low percentages of Ag. In particular, PNC absorption spectra variations are minimal and probably more due to the sampling zone than to the influence of Ag nanoplates.

### 2.2. PNC Effect on Cell Viability and Cytotoxicity

**Figure 5:**
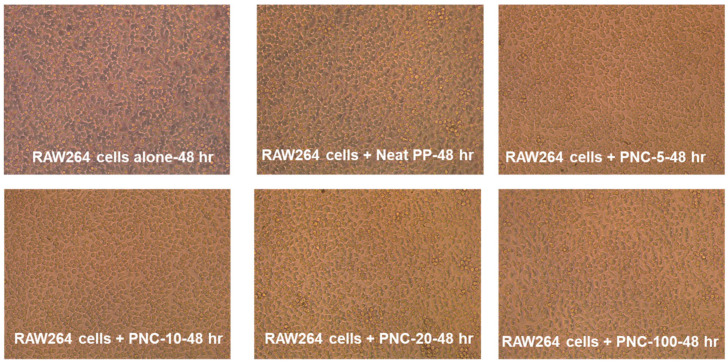
PNC effect on cell viability and cytotoxicity. Murine RAW264 cells (1 × 10^6^ cell/mL) were exposed to 0.5 cm^2^ piece of Neat PP or PNC with increasing concertation of silver (Ag) and incubated for 48 h. RAW264 cell morphology captured with inverted bright microscopy.

The biocompatibility of the formulated PNCs was evaluated by using mammalian RAW264 cells. The data showed that RAW264 cells incubated with NPC containing silver (Neat PP, PNC-5, PNC-10, PNC-20, and PNC-100) retained similar morphology to unexposed cells without aberrant cytoxicity. The effect of PNCs on the viability and morphology of RAW264.7 cells was assessed at 24 h (Figure S1), 48 h (Figure 5), and 72 h (Figure S2). Data revealed no cytotoxic effect of PNCs on mammalian cells. Images also show no reduction in cellular growth and multiplicity.

### 2.3. PNC Effect on Nitric Oxide Release as a Cellular Response

**Figure 6:**
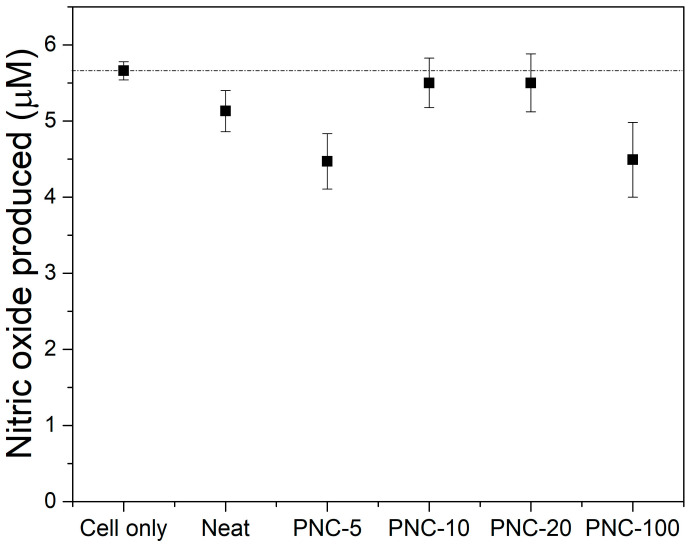
Nitric oxide released from RAW264 macrophages exposed to the different PNC’ samples. Error bars represent standard deviation from the mean of triplicate readout.

To determine whether PNC can induce cellular response in exposed cells, nitric oxide release was used as a surrogate marker for cellular perturbations. Nitric oxide produced by the induction of nitric oxide synthase is a highly reactive radical that converts to a stable nitrite in the supernatants of perturbed cells. In particular, murine RAW264 cells (1 × 10^6^ cell/mL) were exposed to 0.5 cm^2^ piece of PNC alone or PNC with increasing concertation of Ag and incubated overnight. Nitrite accumulation as indicator of nitric oxide release was measured in the supernatants using the Griess assay. Here, we show that PNC formulated alone or with increasing silver concentrations did not induce nitric oxide or nitrite accumulation in the supernatants of exposed RAW264 cells compared to unexposed cells (Figure 6). Consequently, it is evident that PNC does not induce cellular response in mammalian cells, even if a greater randomness of the results is highlighted by the error bars in the presence of Ag. It is noteworthy that these levels of nitric oxide release are the basal levels of production usually observed under any in vitro cellular growth condition without perturbations.

### 2.4. Biofilm Formation on PNC Surfaces

**Figure 7:**
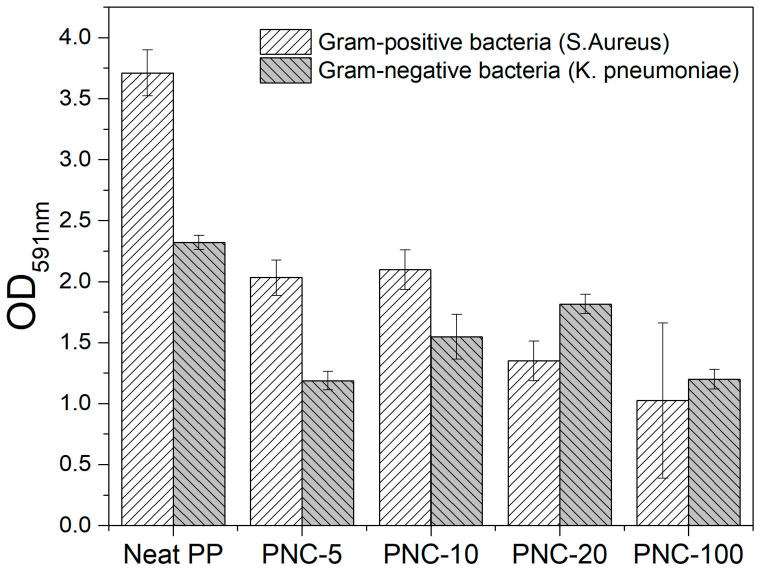
Biofilm formation on samples’ surfaces. Bacterial biofilms were grown on the surfaces of 0.5 cm^2^ pieces with or without Ag and incubated overnight at 37 °C. Biofilm formation by *S. aureus* and by *K. pneumoniae* was measured using 0.1% crystal violet staining. Error bars represent standard deviation from the mean of triplicate readout.

To assess the antibiofilm effect of the formulated PNC, two bacterial pathogens were tested. *Staphylococcus aureus* (*S. aureus*), a Gram-positive bacteria, is a common cause of skin infections and soft tissues, as well as systemic infections. *Klebsiella pneumoniae* (*K. pneumoniae*), a Gram-negative bacteria, is a common cause of various human infections, including healthcare-associated infections. Both pathogens are known for their ability to form biofilms on solid surfaces, which increases the risk of infection. Here, we assessed *S. aureus* and *K. pneumoniae* biofilm formation on the surfaces of Neat PP alone or PNCs with variable Ag wt% content. The data showed that PNCs (0.0023 wt% of Ag or higher concentrations) reduced biofilm formation by *S. aureus* and *K. pneumonia* (Figure 7) when compared to Neat PP.

## 3. Discussion

**Figure 8:**
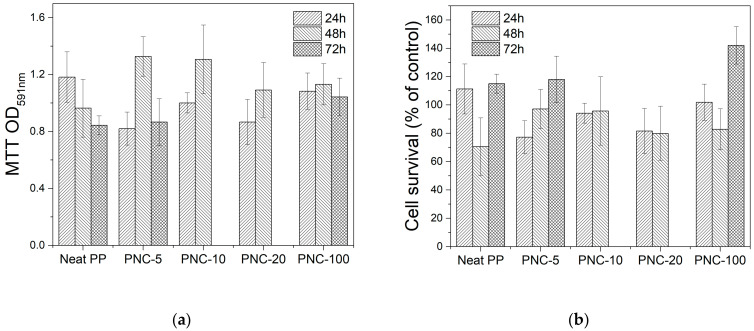
Cell viability of RAW264 cells exposed to PNC material assessed using MTT assay. Error bars represent standard deviation from the mean of triplicate readout: (**a**) MTT assay for the different samples; (**b**) cell survival as a percentage of the control for different samples.

The MTT assay is a sensitive and reliable indicator of the cellular metabolic activity. The results of cell viability measured using the MTT assay with increasing exposure times (24, 48, and 72 h) underlined that no morphological changes or drastic reduction in viability were observed. The observation for 72 h was performed only on PNC-5 and PNC-100 in order to evaluate the effect of the minimum and the maximum of Ag percentages to cell viability. All cells marinated more than 80% viability at 72 h of incubation, which is normal for tissue culture models as cells consume nutrient in the media. Figure 8a,b report the main results for this discussion. Given the fact that these results are generated using in vitro cell culture model with limited number of cells in each well, it is expected that high silver concentration may affect cell variability [35]. However, in this case, the percentages of AgNP and their metallic nature do not affect the cell viability. Investigating the effect of NPC containing silver on tissue interface in an in vivo animal model warrants future investigation.

The data on nitric oxide release suggested that PNC is immunologically inert and does not induce cellular responses in mammalian cells. For a desired nanomaterial to be used in prosthetics, being immunologically inert is crucial to prevent exacerbation of cellular reactions leading unforeseen side effects and complications in vivo [36]. Specifically, prosthetics with direct tissue contact as solid surfaces should not be immunologically active as it may lead to immune rejection and or tissue fibrosis [37]. Therefore, biologically inert nanomaterial is highly desired for use in prosthetics and potential therapeutics.

Bacterial infections are associated with biofilm formation on solid surfaces [38]. The PNC with Ag antibiofilm effect is higher against *S. aureus* compared to *K. pneumoniae* as the latter has a very thick mucoid layer of exopolysaccharides that promotes biofilm formation on solid surfaces. Silver is known to exert antibacterial effect and that explains the reduction in biofilm formation observed here. Silver-coated prosthetics are found to be safe to use in clinical settings with reduced risk of infection [39]. Here, the collected data suggested that PNC with silver could be suitable for future use as prosthetics as it reduced biofilm formation.

**Figure 9:**
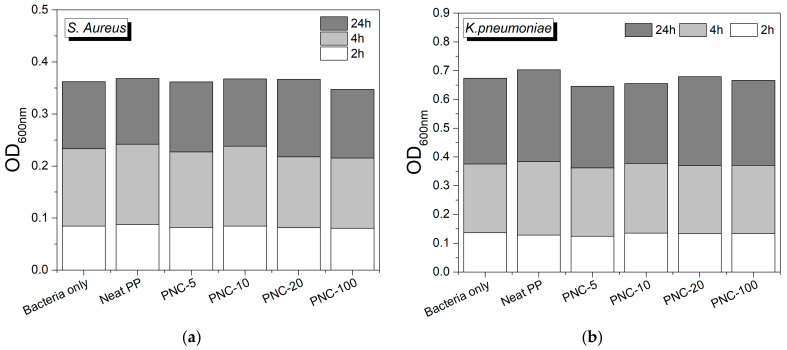
PNC does not inhibit planktonic bacterial growth. Bacterial growth curves of *S. aureus* (**a**) and *K. pneumonia* (**b**) were monitored in the presence and absence of surfaces of 0.5 cm^2^ PNC pieces placed in designated wells and incubated for 24 h at 37 °C.

Although the tested PNC with silver reduced biofilm formation, it did not affect the ability of planktonic bacteria to grow, indicating that in case of infection, PNC does not exert antibacterial effect against growing planktonic *S. aureus* (Figure 9a) and *K. pneumonia* (Figure 9b). The results show that planktonic bacteria were able to grow in the presence of PNC with increasing silver concentrations as assessed at 2 h, 4 h, and 24 h of bacterial growth. These experiments were performed ex vivo in 24-well plates with dense starting bacterial inoculums that allow rapid growth of bacteria. However, with increased incubation time, a significant depletion of nutrients occurs with bacterial growth; hence, we may not observe the effect of silver as it becomes very diluted compared to the large number and density of bacterial cells. Experiments with lower bacterial inoculum may reflect the effect of silver on planktonic bacterial growth, which warrant further investigation.

Moreover, AgNP tends to base its effect on oxidative dissolution that proceeds to completion under oxic conditions. However, the speed and extent of dissolution depend on several factors. A specific study on this aspect will be necessary to investigate the stability and rate of dissolution of particles.

Specifically, this study is the first stage of testing the fabricated PNC material biocompatibility and it was conducted in an in vitro tissue culture system. The future plan is to design 3D-printed prosthetics using this PNC material which will be then tested in in vivo animal models as well as in in vitro tissue culture models.

## 4. Materials and Methods

### 4.1. Polypropylene Nanocomposite (PNC) Samples’ Production

The polymer matrix used for the production of PNCs is an injection-molding-grade polypropylene (PP) (Moplen HC500N, Lyondellbasell Europe). This is a commercial polypropylene in the form of pellet widely used in common industrial applications as biomedical, packaging, nonwoven fabrics, etc. The PP used had a nominal density of 0.9 g/cm^3^ and an average pellet size in the range of 2.5–4.8 mm. Nanocomposite reinforcement was 99.99% pure silver (Ag). This was used in the form of a rectangular target (300 × 125 mm^2^) for PVD (physical vapor deposition) sputtering.

The PVD deposition of Ag on PP pellets was performed using a magnetron sputtering physical deposition system of MITEC s.r.l. with 400 W as DC power input, 20 min as deposition time, and 29 rpm of rotation speed of the pellets during the sputtering phase. These parameters allow to deposit a percentage of Ag of 0.046 ± 0.001% on the pellets’ surface. The molding step was carried out using an electric press (Fanuc Roboshot S-2000i 50B) of 50 t. Neat molded square plates were produced using only PP pellets without coating, while PNCs square plates were molded using nanocoated PP pellets. The pellet’s nanocoating was fragmented in the screw by the shear forces and distributed during molding. Different PNCs were molded using different mixing ratio between PP pellets without coating and nanocoated PP pellets. Accordingly, the PNCs samples had within them metal Ag particles content up to 0.046 wt% that vary in surface extension but have a thickness of about 25.3 ± 0.5 nm. Thus, the nanometric thickness allows us to consider the particles as nanofillers. Thermal and mechanical properties of the PNCs were reported by authors in previous works [30].

The roughness measurements were carried out with a surface analyzer by Taylor Hobson (Talysurf CLI 2000) in contact mode with an inductive probe. For each sample, 50 profiles were acquired to cover a representative sample area of 15 × 5 mm^2^. The acquired profiles were analyzed using Talysurf 3.1 software, with a Gaussian filter of 0.8 mm for the roughness measurements, in order to represent the surface morphology of the PNCs.

The AFM measurements were performed using a Nanosurf FlexAFM with C3000 controller. Measurements were carried out in tapping mode with Dyn190Al probes (spring constant 48 N/m). The resolution of 512 × 512 data points at 0.1 Hz per image was used to collect the images.

The infrared spectrum of the PNCs was performed on an FT/IR-4X (Jasco, Mary’s Court, Easton, MD 21601, USA) spectrometer in a range of 400–7000 cm^−1^ (KRS-5). It was used coupled with the ATR-PRO4X (ZnSe prism) single reflection accessory with an angle of incidence of 45° and a contact area with a diameter of 2.5 mm. Each spectrum was acquired 10 times with a wavenumber resolution of 0.4 cm^−1^ and accumulation of 32 scans.

### 4.2. Cell Viability Measurement Using MTT

RAW264.7 cells (obtained from ATCC, Manassas, VA, USA) were freshly grown in DMED tissue culture medium containing 10% FBS and 1% penicillin–streptomycin (purchased from Thermo-Fisher Scientific, Waltham, MA, USA). Cells were harvested using cell scrapers (purchased from Thermo-Fisher) and adjusted to 1 million cell per ml then transferred to a 24-well plate and incubated overnight in the absence and presence of the nanocomposites with different increasing Ag concentrations (Neat, 5%, 10%, 20%, and 100%). The tested PNC was cut into 0.5 cm^2^ sized pieces and each sample was tested in duplicate wells per experiment. Cells cultured without the nanocomposite were used as a control. The next day, cells were harvested using cell scrapers and transferred to a 96-well plate to be tested for viability using MTT assay. From the 5 mg/mL of thiazolyl blue tetrazolium bromide (purchased from Sigma, St. Louis, MO, USA) dissolved PBS, 15 μL was added to the cells and incubated for 1.5 h at 37 °C. After one PBS wash, 150 μL of DMSO was added to dissolve the dye. The wavelength was measured at 591 nm [40]. For longer time exposure of mammalian RAW264.7 cells to PNCs, similar experiments were conducted at 24, 48, and 72 h in the presence of the nanocomposites. First, the nanocomposites were washed twice with DMEM (10% FBS and 1% penicillin–streptomycin), then placed in 12-well plates. A total of 3 plates was used for the experiment, with one plate for each time point (24, 48, and 72 h). RAW264.7 cells were seeded by adding 3 mL of RAW264.7 cell suspension into the wells and the plates were incubated at 37 °C and 5% CO_2_ in presence and absence of PNCs as mentioned above. Images of RAW264.7 cells were captured using an inverted microscope with camera at 24, 28, and 72 h of incubation with PNCs for multiplicity and morphological changes observation.

### 4.3. Nitric Oxide

Nitric oxide levels resulting from incubating RAW264.7 cells overnight with nanocomposites formulated with different increasing silver concentrations (Neat, PNC-5, PNC-10, PNC-20, and PNC-100) were measured. This was carried out by preparing a mixture of sulfanilamide dissolved in 1% orthophosphoric acid and N-(1-Naphthylethylenediamine) dihydride (purchased from Sigma-Aldrich, St. Lois, MO, USA) in a 1:1 ratio as previously described [41]. In a microtiter plate, 100 μL of the mixture was added to 100 μL of the RAW264.7 supernatants and read at 540 nm. Serially diluted nitrite ions were used as standards and PBS was used as a blank.

### 4.4. Growth Curve Measurements

Bacterial growth for *S. aureus* (ATCC strain number 23235) and *K. pneumonia* (ATCC strain number BAA-2342) was measured in the presence and absence of the nanocomposite material using standard turbidity. Bacterial cells were cultured overnight on nutrients agars at 37 °C (purchased from Thermo-Fisher, USA). The next day, the colonies were suspended in tryptic soy broth (TSB, also purchased from Thermo-Fisher) to prepare an inoculum with an O.D. of 0.1. The inoculum was diluted in 2 mL TSB, making a 1:20 ratio and incubated with increasing silver concentrations (Neat, PNC-5, PNC-10, PNC-20, and PNC-100). At each time point, 100 μL of the broth in the experimental plate was taken into a microtiter plate to measure the absorbance at 600 nm. Optical density measurements were taken at 3 different time points (after 2, 4, and 24 h).

Biofilm formation: The biofilm forming on the nanocomposites was measured after the overnight incubation for the growth curve by crystal violet staining. The nanocomposites were placed in a new plate and had 0.1% of crystal violet added to them. After 10 to 15 min of room temperature incubation, the crystal violet was washed out by water and 30% acetic acid was added to dissolve the dye. The incubation was repeated and 100 μL of the dissolved stain was taken into a new plate and the wavelength of 550 nm was read. The 30% acetic acid was used as a blank as previously described [42].

Statistical analysis: The statistical analysis was carried out on the cell viability measurements, on the nitric oxide production, on the bacterial growth, and film formation. It was performed using Microsoft Excel software. Student’s *t*-test analysis was conducted. *p* values less than 0.05 were considered significant.

## 5. Conclusions

In this work, results of cytotoxicity and antibiofilm activity of different composites with polypropylene (PP) matrix, and Ag nanoplates as an antimicrobial agent, were discussed. The innovative nanocoating fragmentation process was used for samples fabrication. Ultra-low filling content of nanoplates were obtained without affecting the surface morphology of PNCs. The PNC containing metallic Ag possessed antibiofilm activity although it did not inhibit regular planktonic bacterial growth. Moreover, the PNC was not cytotoxic to mammalian cells and did not induce significant immune response. The discussed results suggest that PNC with Ag could be suitable for future use as prosthetics as it reduced biofilm formation; in particular, it seems to be sufficient to use a low percentage of Ag, such as in PNC-5. In particular, a future plan is to design 3D-printed prosthetics using this PNC-5 material with silver coating which will be then tested in in vivo animal models as well as in in vitro tissue culture models.

## Data Availability

Data are contained within the article or supplementary material. The data presented in this study are available in [29,30,31].

## Supplementary Materials

The following are available online at https://www.mdpi.com/article/10.3390/antibiotics12050924/s1, Figure S1: PNC effect on cell viability and cytotoxicity. Murine RAW264 cells (1 × 106 cell/mL) were exposed to 0.5 cm^2^ piece of Neat PP or PNC with increasing concertation of silver (Ag) and incubated for 24 hr. RAW264 cell morphology captured with inverted bright microscopy; Figure S2: PNC effect on cell viability and cytotoxicity. Murine RAW264 cells (1 × 106 cell/mL) were exposed to 0.5 cm^2^ piece of Neat PP or PNC with increasing concertation of silver (Ag) and incubated for 72 hr. RAW264 cell morphology captured with inverted bright microscopy.
