# Supplementary material for: Cytotoxicity and Antibiofilm Activity of Silver-Polypropylene Nanocomposites
Source: Antibiotics (Basel). 2023 May 17;12(5):924. doi: 10.3390/antibiotics12050924 (PMC10215392; doi:10.3390/antibiotics12050924)
Supplement: Supplementary file 1 [file antibiotics-12-00924-s001.zip › antibiotics-2329557-supplementary.pdf]

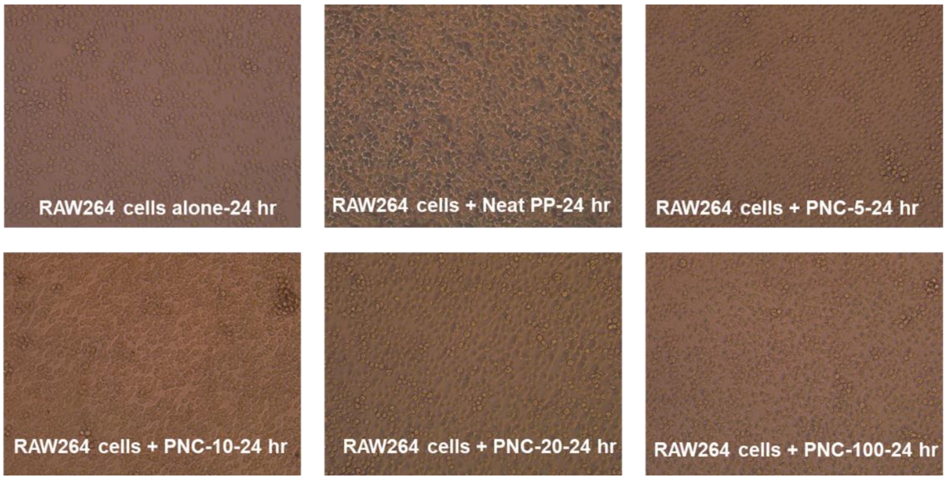

Figure S1. PNC effect on cell viability and cytotoxicity. Murine RAW264 cells (1x 10^6 cell/ml) were exposed to 0.5 cm^2 piece of Neat PP or PNC with increasing concertation of silver (Ag) and incubated for 24hr. RAW264 cell morphology captured with inverted bright microscopy.

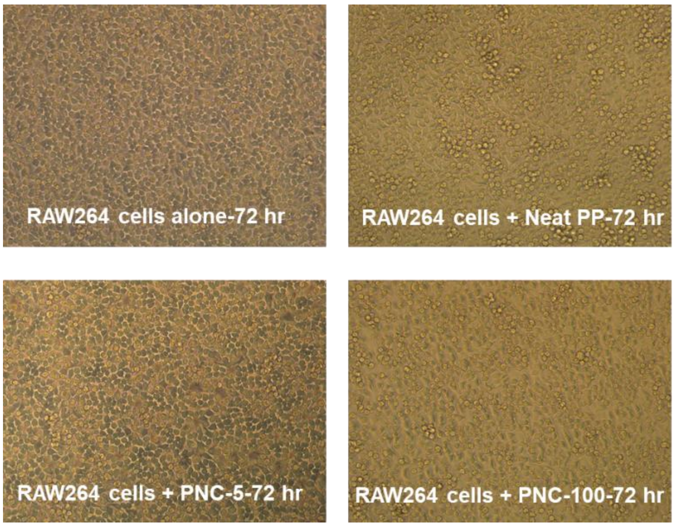

Figure S2. PNC effect on cell viability and cytotoxicity. Murine RAW264 cells (1x 10^6 cell/ml) were exposed to 0.5 cm^2 piece of Neat PP or PNC with increasing concertation of silver (Ag) and incubated for 72hr. RAW264 cell morphology captured with inverted bright microscopy.
